# Supplementary figures and images for: A Hidden Markov Model Approach for Simultaneously Estimating Local Ancestry and Admixture Time Using Next Generation Sequence Data in Samples of Arbitrary Ploidy
Source: PLoS Genet. 2017 Jan 3;13(1):e1006529. doi: 10.1371/journal.pgen.1006529 (PMC5242547; doi:10.1371/journal.pgen.1006529)

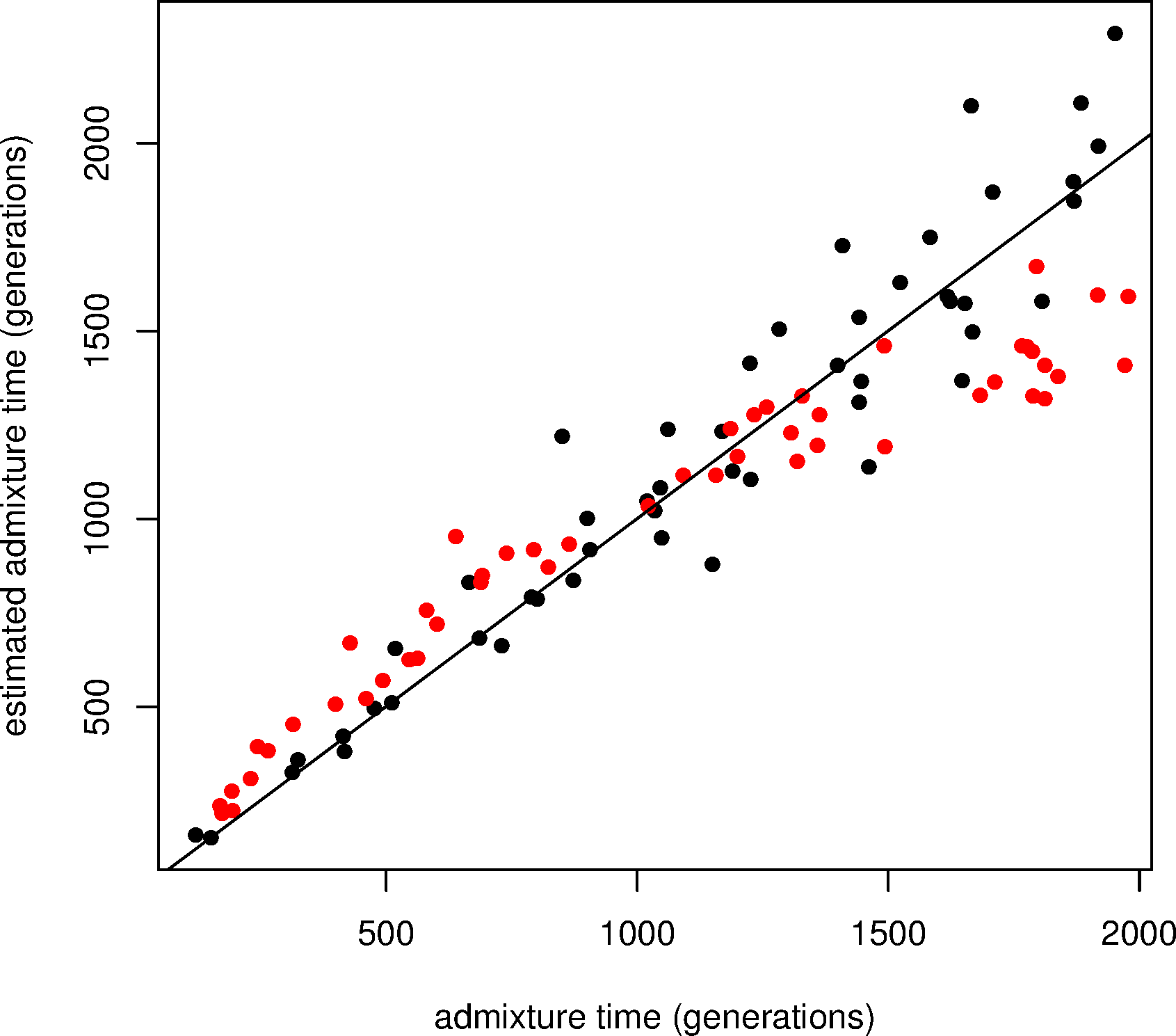

Supplement: S1 Fig — Simulations were conducted using an ancestry proportion of 0.25 and population size of 10,000 hermaphroditic individuals. (TIF) [file pgen.1006529.s001.tif]

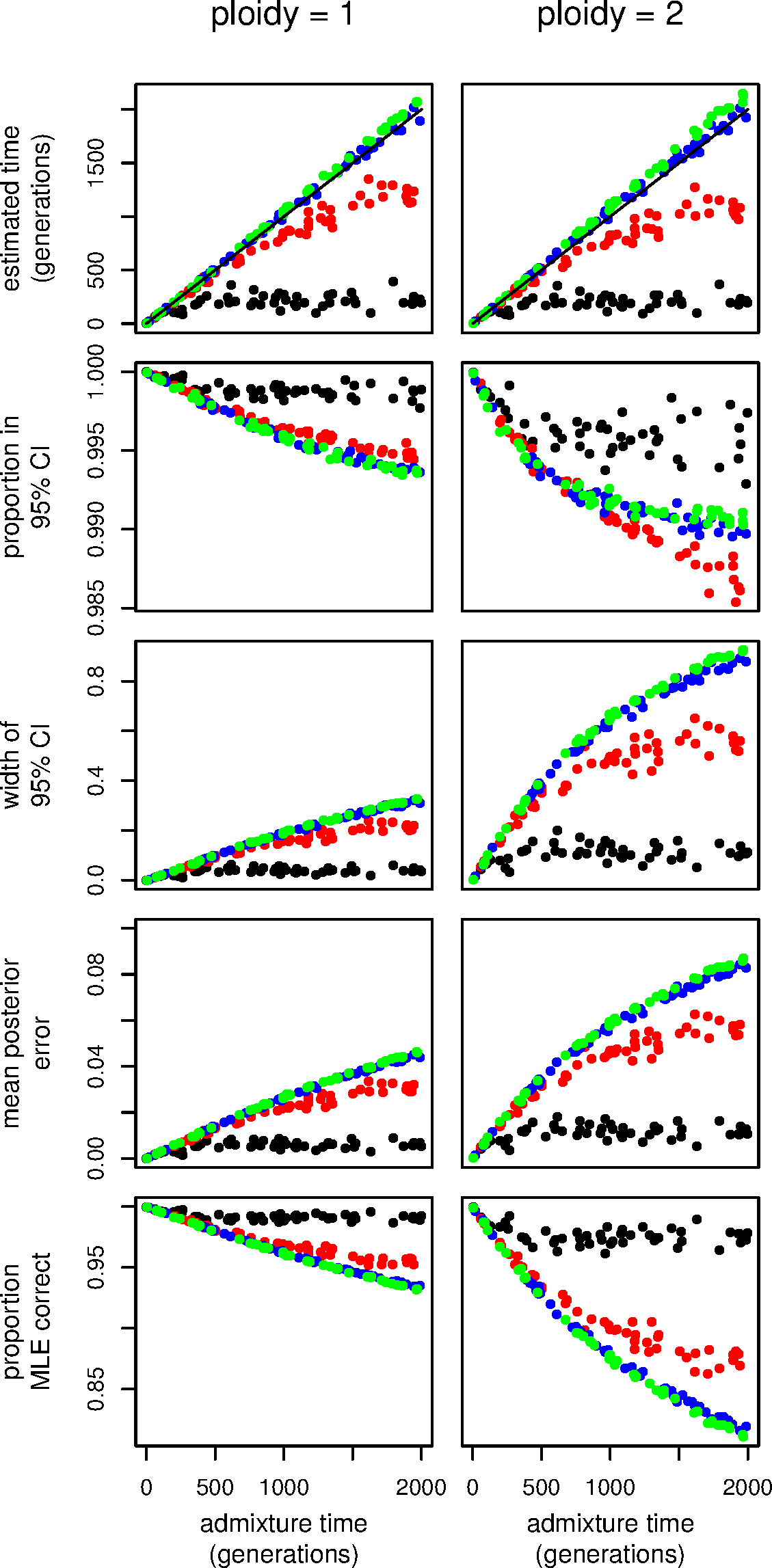

Supplement: S2 Fig — All LAI was conducted assuming the true population size was 10,000. Simulated population sizes were 100 (black), 1,000 (red), 10,000 (blue) and 100,000 (green). Ploidy 1 on the right, ploidy 2 on the left. From top to bottom, rows are the estimated time of admixture, the proportion of sites where the true state is within the 95% credible interval, the width of the 95% credible interval, the mean posterior error, and the proportion of times that the maximum likelihood estimate is equal to the true state. For all simulations, the ancestry proportion was equal to 0.5. (TIF) [file pgen.1006529.s002.tif]

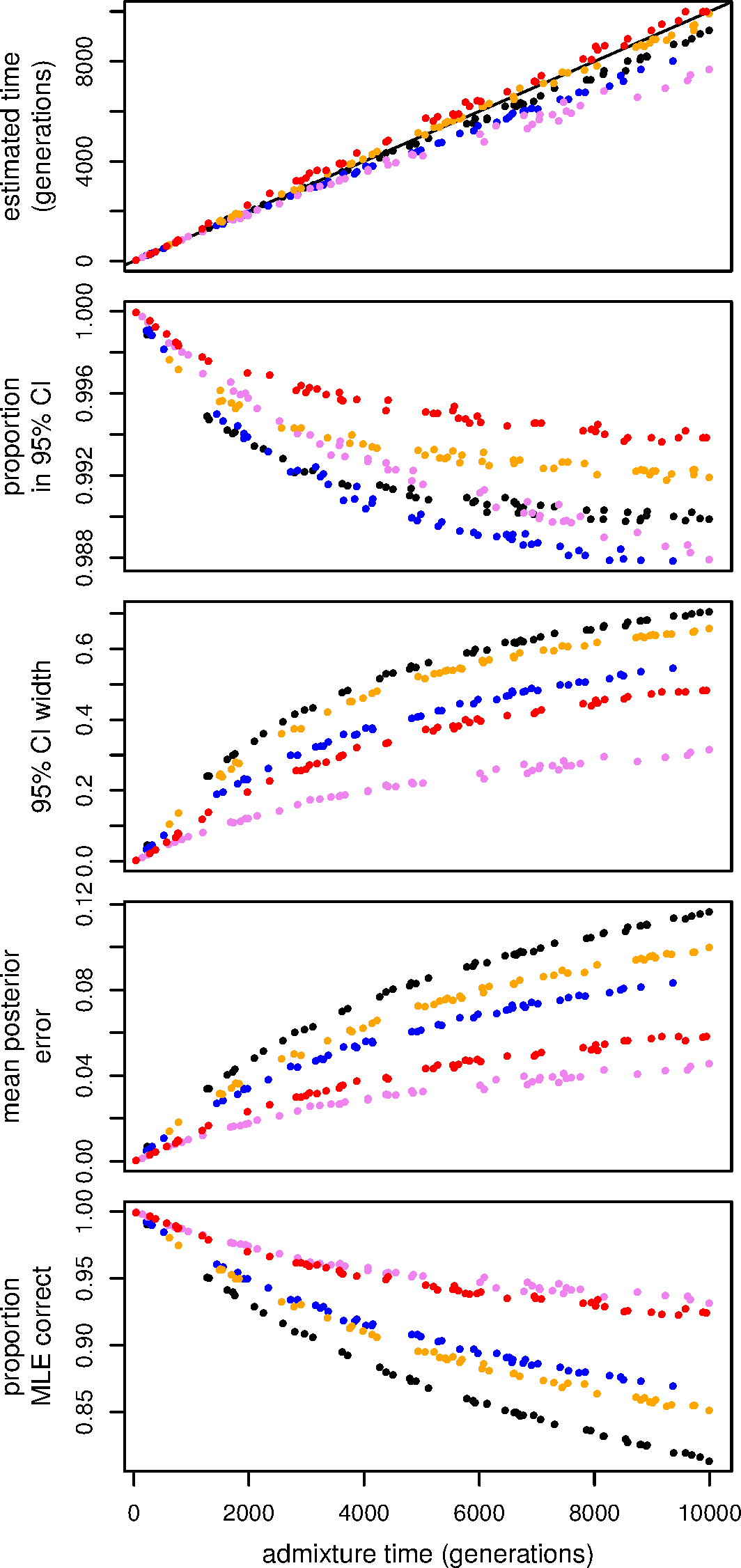

Supplement: S3 Fig — Here, ancestry proportions are 0.5 (black), 0.25 (blue), 0.1 (violet), 0.75 (orange) and 0.9 (red). From top to bottom, statistics plotted are estimated time, the proportion of sites where the true ancestry frequency is within the 95% credible interval, the mean 95% credible interval width, mean posterior error, and the proportion of times that the maximum likelihood estimate is correct. (TIF) [file pgen.1006529.s003.tif]

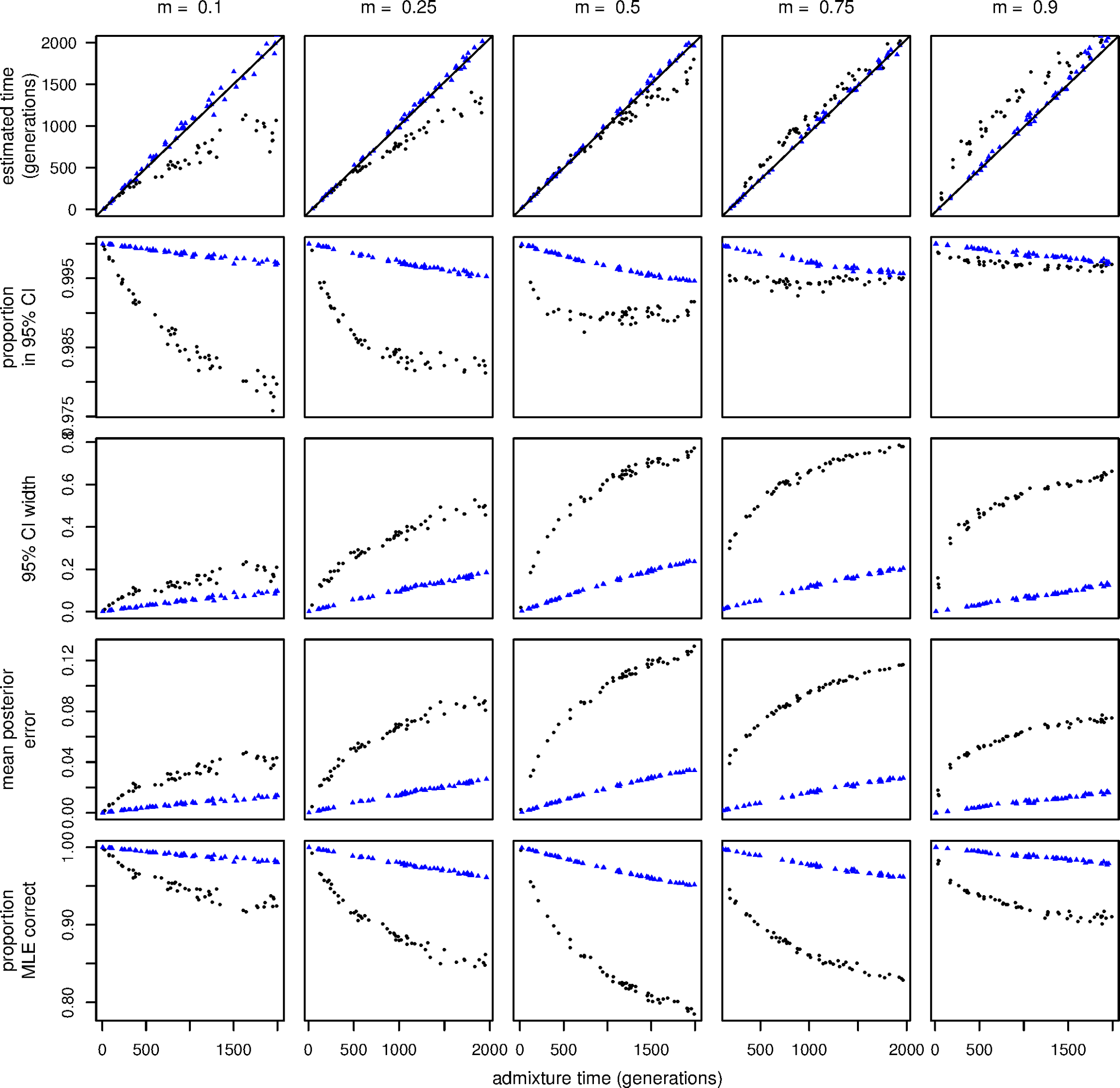

Supplement: S4 Fig — Here, we compare reference panels of size 100 (blue) with reference panels of size 10 (black). From left to right, ancestry proportions are 0.1, 0.25, 0.5, 0.75 and 0.9. From top to bottom the plotted statistics are estimated time, proportion in the 95% credible interval, the average width of the 95% credible interval, the mean posterior error, and the proportion of sites where the maximum likelihood ancestry estimate is correct. (TIF) [file pgen.1006529.s004.tif]

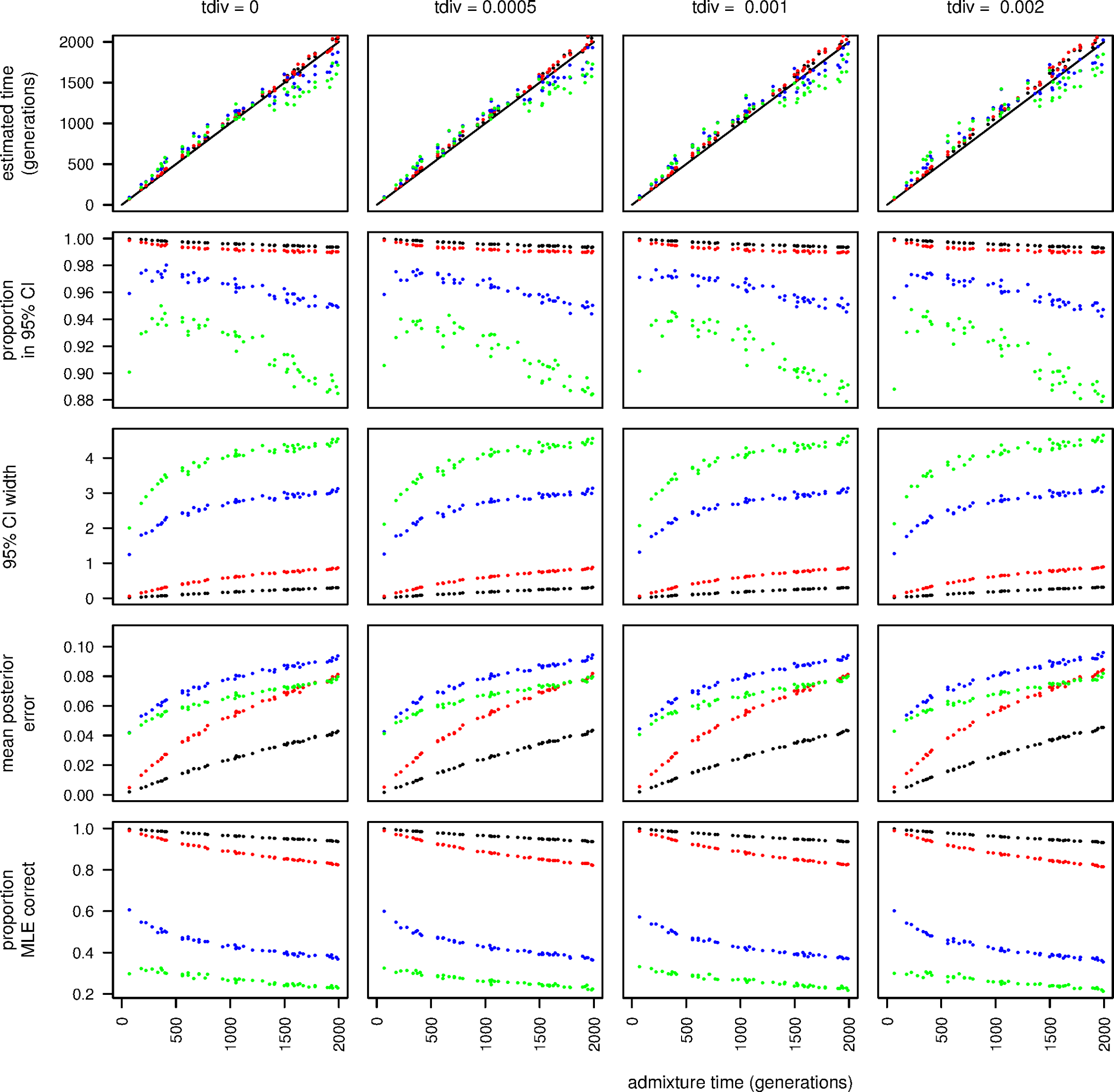

Supplement: S5 Fig — In columns are divergence times between ancestral populations (in units of 4Ne) of 0, 0.0005, 0.001, 0.002. From top to bottom the plotted statistics are estimated time, proportion in the 95% credible interval, the average width of the 95% credible interval, the mean posterior error, and the proportion of sites where the maximum likelihood ancestry estimate is correct. (TIF) [file pgen.1006529.s005.tif]

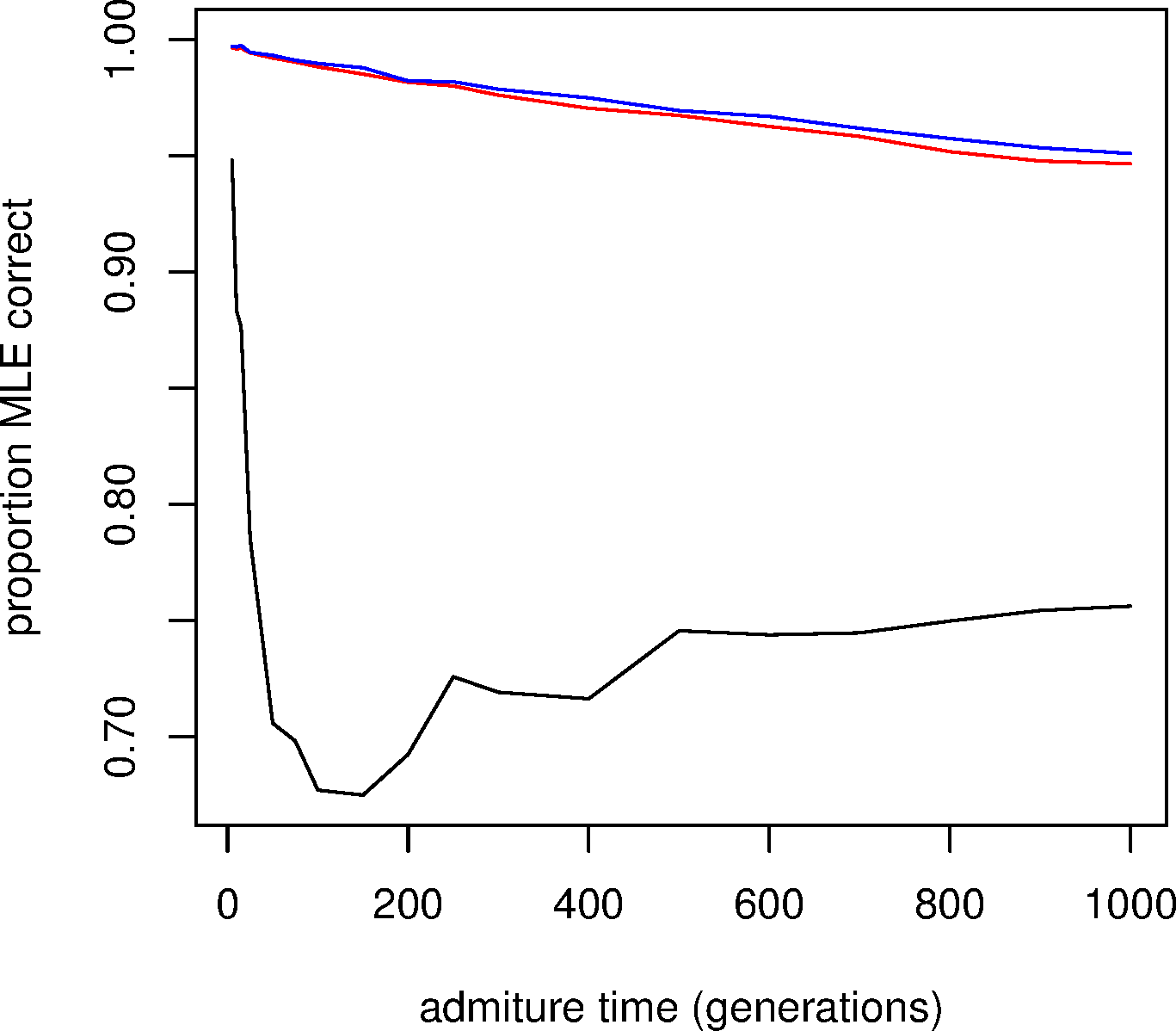

Supplement: S6 Fig — WinPop was run with default parameters (black), and with LD pruned in the ancestral populations, but not in the admixed population (red). Our method was run with default parameters (blue), but with the time since admixture and correct ancestry proportion supplied to our program as these parameters are required by WinPop. (TIF) [file pgen.1006529.s006.tif]

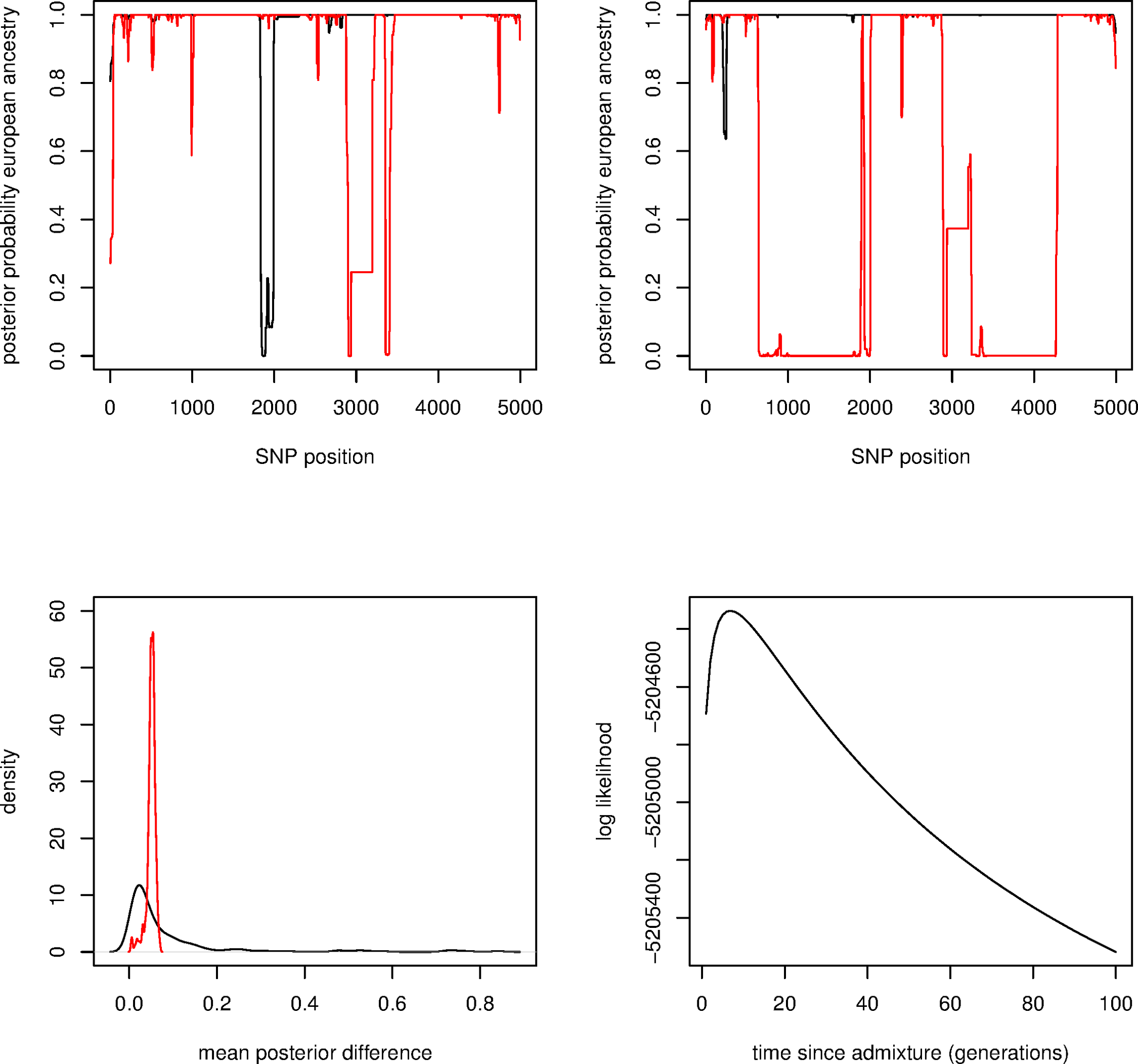

Supplement: S7 Fig — The posterior probability estimated using RFMix of European ancestry at a given site in the genome assuming t = 5 (black) and assuming t = 20 (red) for a sample representative of the average difference (top left) and a more extreme example (top right). The distribution of differences in mean Inuit ancestry for all samples (bottom left) using RFMix. The log likelihood of each time since admixture as computed using our method (bottom right), which shows a clear optimum at 6–7 generations since admixture. All analyses were restricted to SNPs on chromosome 10. (TIF) [file pgen.1006529.s007.tif]

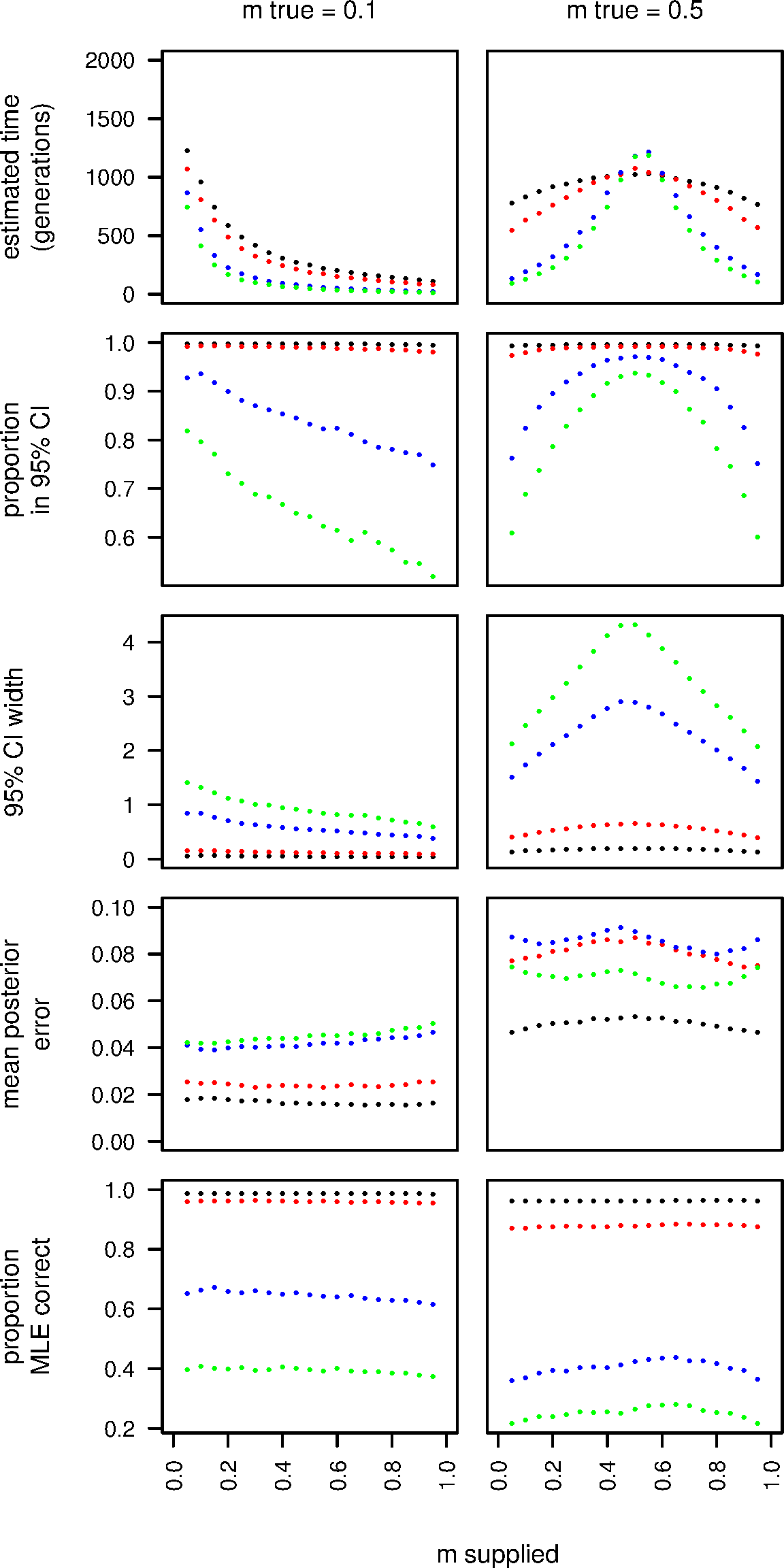

Supplement: S8 Fig — On the left, true m is 0.1and on the right true m is 0.5. Supplied m varies across 0.05 to 0.95. From top to bottom, the plotted statistics are estimated t, proportion in the 95% confidence interval, mean 95% confidence interval width, mean posterior error and the proportion of sites where the maximum likelihood estimate is correct. All plots include ploidy one (back), ploidy two (red), ploidy ten (blue), and ploidy twenty (green). (TIF) [file pgen.1006529.s008.tif]

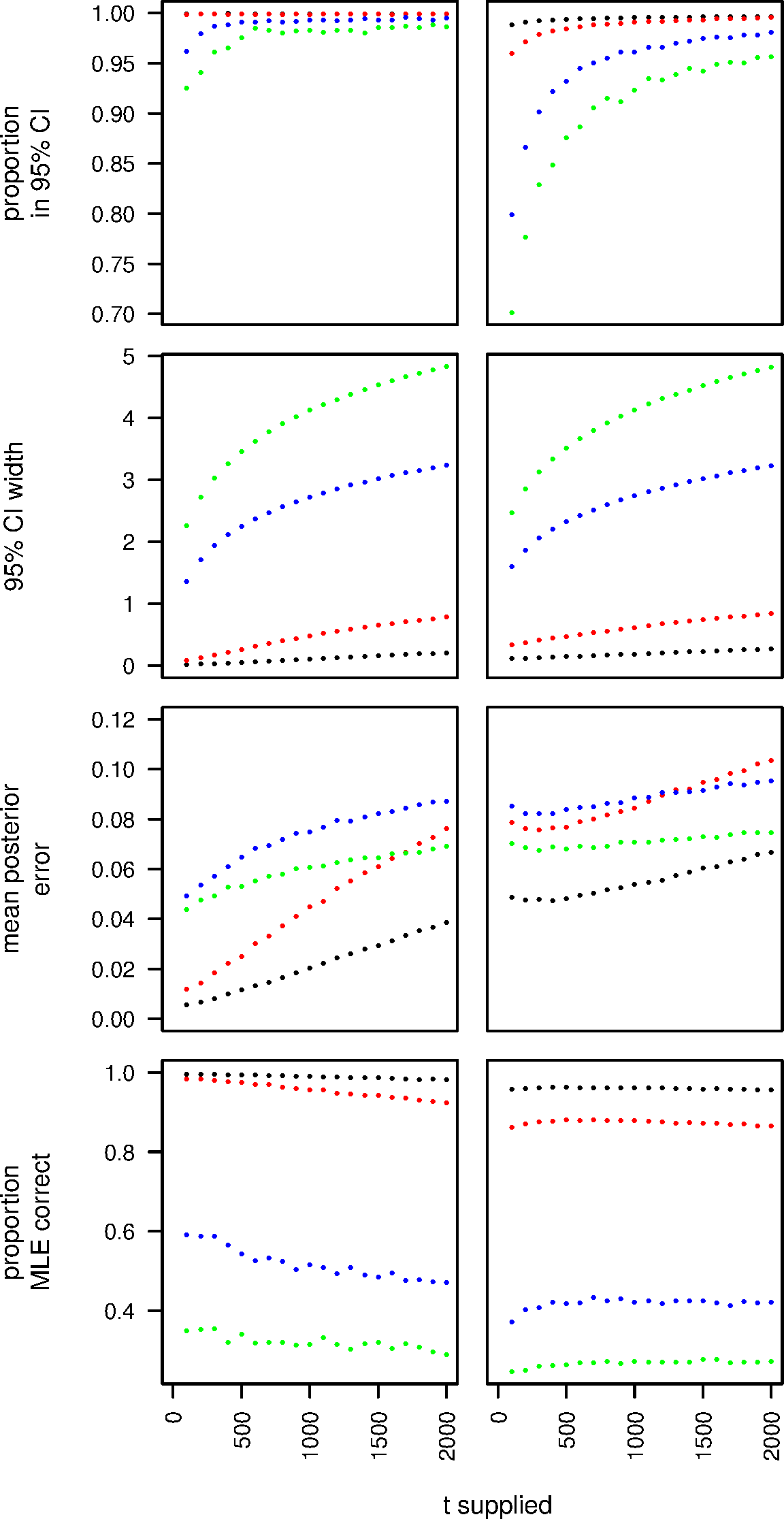

Supplement: S9 Fig — On the left, true t is 100 and on the right true t is 1000. Supplied t varies across 100 to 2000 generations. From top to bottom, the plotted statistics are proportion in the 95% confidence interval, mean 95% confidence interval width, mean posterior error and the proportion of sites where the maximum likelihood estimate is correct. All plots include ploidy one (back), ploidy two (red), ploidy ten (blue), and ploidy twenty (green). (TIF) [file pgen.1006529.s009.tif]

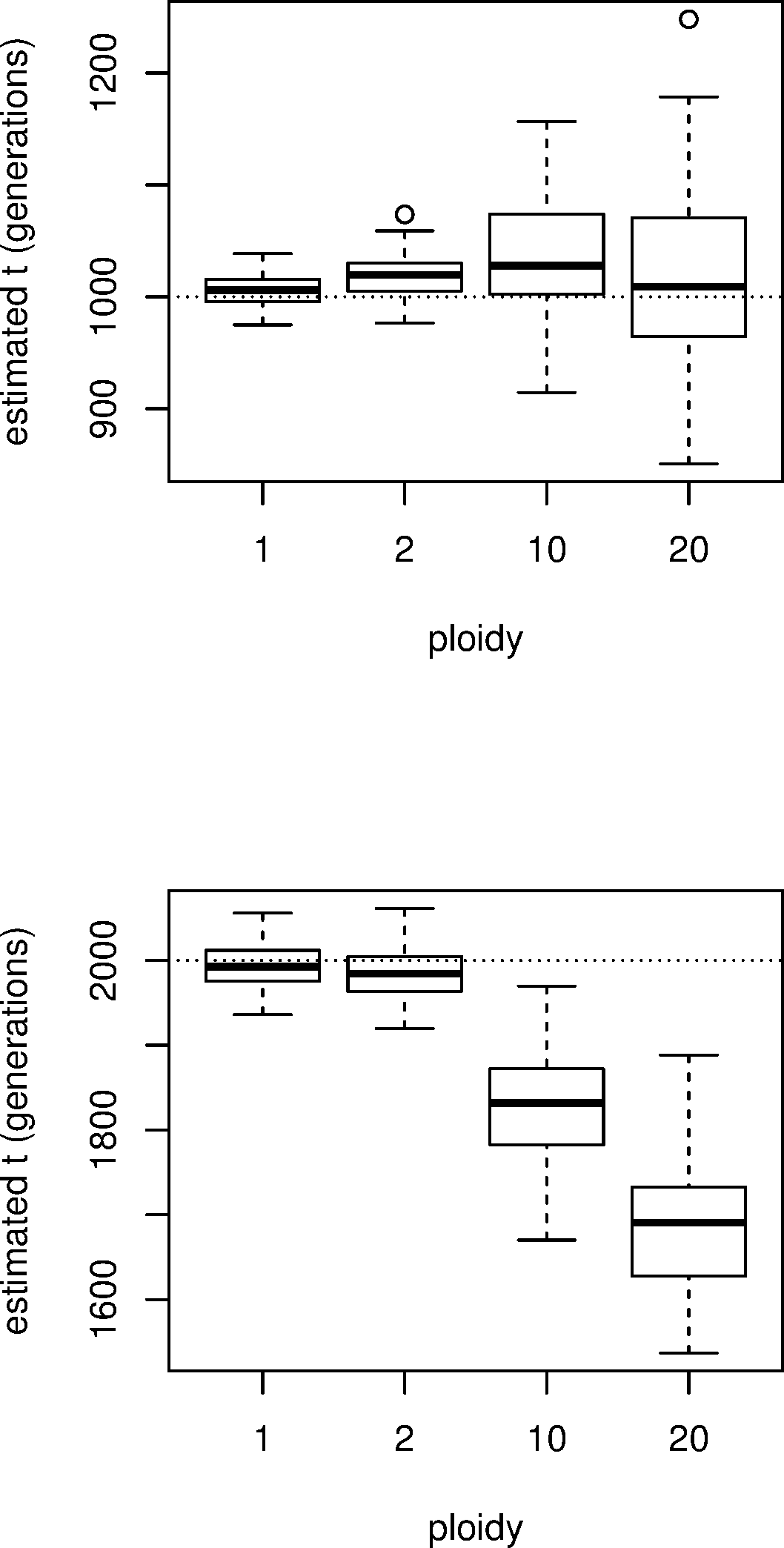

Supplement: S10 Fig — From left to right, sample ploidies are 1, 2, 10, and 20. For both simulations, m = 0.5. (TIF) [file pgen.1006529.s010.tif]
